# Using graphic modelling to identify modifiable mediators of the association between area-based deprivation at birth and offspring unemployment

**DOI:** 10.1371/journal.pone.0249258

**Published:** 2021-03-31

**Authors:** James Bogie, Michael Fleming, Breda Cullen, Daniel Mackay, Jill P. Pell

**Affiliations:** 1 Institute of Health and Wellbeing, University of Glasgow, Glasgow, United Kingdom; 2 Institute of Health and Wellbeing, Academic Centre, Gartnavel Royal Hospital, Glasgow, United Kingdom; University of Oslo, NORWAY

## Abstract

**Background:**

Deprivation can perpetuate across generations; however, the causative pathways are not well understood. Directed acyclic graphs (DAG) with mediation analysis can help elucidate and quantify complex pathways in order to identify modifiable factors at which to target interventions.

**Methods and findings:**

We linked ten Scotland-wide databases (six health and four education) to produce a cohort of 217,226 pupils who attended Scottish schools between 2009 and 2013. The DAG comprised 23 potential mediators of the association between area deprivation at birth and subsequent offspring ‘not in education, employment or training’ status, covering maternal, antenatal, perinatal and child health, school engagement, and educational factors. Analyses were performed using modified g-computation. Deprivation at birth was associated with a 7.3% increase in offspring ‘not in education, employment or training’. The principal mediators of this association were smoking during pregnancy (natural indirect effect of 0·016, 95% CI 0·013, 0·019) and school absences (natural indirect effect of 0·021, 95% CI 0·018, 0·024), explaining 22% and 30% of the total effect respectively. The proportion of the association potentially eliminated by addressing these factors was 19% (controlled direct effect when set to non-smoker 0·058; 95% CI 0·053, 0·063) for smoking during pregnancy and 38% (controlled direct effect when set to no absences 0·043; 95% CI 0·037, 0·049) for school absences.

**Conclusions:**

Combining a DAG with mediation analysis helped disentangle a complex public health problem and quantified the modifiable factors of maternal smoking and school absence that could be targeted for intervention. This study also demonstrates the general utility of DAGs in understanding complex public health problems.

## Introduction

Socio-economic deprivation is a risk factor for a wide range of health indicators from birth through adolescence [1–6], as well as poorer educational outcomes [2, 7–11]. Deprivation can perpetuate between generations. In 2017, the Scottish government reported a 14·9 percentage point difference in the rate of participation in employment, education or training between pupils from the most deprived areas compared to the least deprived [12]. Stewart et al. demonstrated that, in a Scottish school leavers cohort, increased deprivation at birth was associated with poorer attainment and that poorer attainment on leaving school was associated with increased unemployment, however the analyses were not adjusted for confounders [13].

Elucidating the pathways through which parental deprivation predisposes to offspring deprivation could help to identify modifiable factors that, if tackled, could break the current cycle of ‘inherited’ health inequalities. Constructing a directed acyclic graph (DAG) has been recommended to guide analyses of neighbourhood health effects [14] and to understand bias and confounding [15–17]. When coupled with gformula analysis, they can estimate the proportion of an effect that is explained by a mediator. To the best of our knowledge, this approach has not previously been used to identify factors that mediate the association between deprivation at birth and offspring ‘not in education, employment or training’ (NEET).

Scotland is well placed to undertake this type of research due to its large number of high-quality, national administrative datasets that can be linked at an individual level. This study used record linkage of routinely collected data to construct and analyse a graphical model of the factors that mediate the association between parental and offspring deprivation; measured by area-based deprivation at birth and offspring NEET respectively.

## Methods

### Databases and inclusion criteria

Individual-level data were linked from six Scotland-wide administrative health databases, held by the Information Services Division of the National Health Service (NHS), and four Scotland-wide education databases held by the Scottish Exchange of Educational Data. The linkage process has been described previously [13, 18]. The Scottish Morbidity Record (SMR) collects data on admissions to hospital including dates of admission and discharge and International Classification of Diseases (ICD) diagnostic codes for acute (SMR01) and psychiatric (SMR04) hospitals and neonatal (age 0–28 days) units (SMR11). SMR02 collects additional antenatal, obstetric and neonatal data for admissions to maternity hospitals. The Prescribing Information System records data on all medications dispensed in the community. The Child Health Surveillance Programme Pre-School Dataset collects information obtained by health visitors on developmental milestones and feeding. The School Pupil Census, conducted annually in September, collects data on all children attending Scottish local authority-run primary, secondary and special schools. This includes any record, and type, of special educational need and whether a child is looked after by the care system. The Scottish Qualifications Authority collates exam results for all children and the school-leavers’ database collects information on school leaver status six months after leaving school. Data on school absences and exclusions are collected prospectively and appended to the School Pupil Census at the end of the relevant school year.

Study inclusion was restricted to singleton children who attended Scottish schools between 2009 and 2013 inclusive and who were born in Scotland. Since pupils are permitted to leave school between the ages of 16 to 18 years, study participants were born between 1991 and 1998.

### Exposure, outcome and confounder variables

The exposure was area-based deprivation at birth; derived from the postcode of residence recorded on SMR02 at the time of delivery using the 2012 Scottish Index of Multiple Deprivation. The Scottish Index of Multiple Deprivation is a measure of relative deprivation derived for all postcodes of residence across Scotland. It is calculated from neighbourhood-level measures of 38 indicators across seven domains: income; employment; health; education; housing; geographic access; and crime. General population quintiles were derived and dichotomised into the most deprived quintile and the four less deprived quintiles. Since it is common to reside in student accommodation or with parents for several years after leaving school, postcode of residence was not considered to be a good measure of the offspring’s personal socioeconomic status. Therefore, the outcome of offspring not in education, employment or training was used instead. School leaver status was dichotomised into unemployed versus in further/higher education, training or employment six months after leaving school. Ethnicity and sex were treated as potential confounding variables because they did not lie on any of the causal pathways.

### Directed acyclic graph

A directed acyclic graph (DAG) was used to visually depict assumptions about causal relationships. Construction of the DAG began prior to data analyses. All possible pairs of variables were systematically assessed applying known and/or published evidence of causal relationships. Unclear associations were discussed by the research team, with input from two Consultant Neonatologists where appropriate.

**Fig 1:**
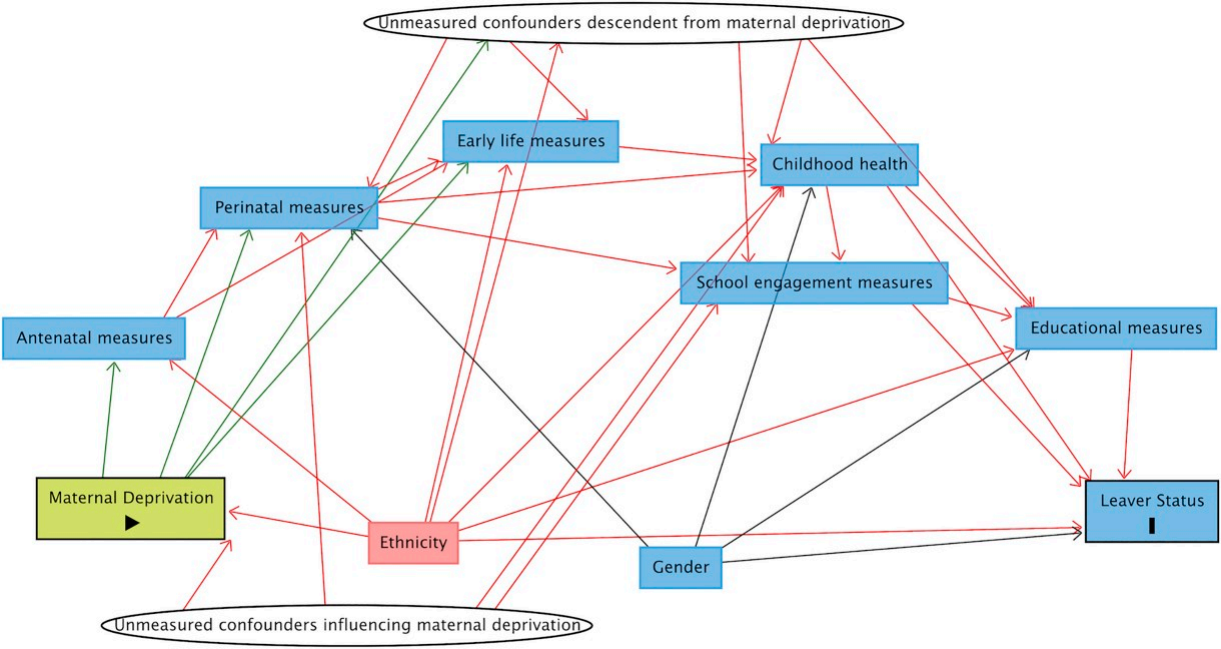
Simplified directed acyclic graph. Directed acyclic graph showing causal assumptions between grouped nodes.

The DAG was created using Dagitty software [19]. Each node represented a variable of interest. Arrows between nodes denoted causal relationships, pointing from cause to effect, and included even weak assumptions of causal relationships and causal relationships present only in a sub-group. Lack of an arrow denoted confidence that no causal relationship existed based on evidence [20]. The full DAG (S1 Fig) was used in the analyses but a simplified version, with collapsed variables, is also provided (Fig 1) for ease of interpretation.

Antenatal nodes included self-reported smoking during pregnancy, maternal age (<25, 25–29, 30–34, or >34 years), and parity (nulliparous, parous, or multiparous). Perinatal nodes included mode of delivery (assisted versus non-assisted) and APGAR score at 5 minutes (1–3, 4–6, or 7–10). Gestational age and birth weight were used to derive sex-, gestation-specific birthweight centiles as a measure of intrauterine growth restriction. Health visitors recorded developmental milestones across four domains (gross motor, hearing and communication, manipulative skills, and social and behavioural) as normal, abnormal, doubtful, or incomplete. Children classified as doubtful or abnormal for any domain at the 6–8 week, 8–9 month, 22–24 month, or 39–42 month assessments were then coded as having milestone concerns. Due to large amounts of missing data, we were unable to analyse nodes in the DAG for maternal body mass index, drug and alcohol consumption during pregnancy, and breastfeeding, therefore these were marked as unmeasured.

Neonatal records (SMR11) were used to ascertain congenital anomalies (ICD9 740–758 or ICD10 Q00-Q99). Hospital admissions were coded as the total number of admissions to acute or psychiatric units recorded on SMR01 and SMR04 respectively. Admissions secondary to trauma were identified using ICD9 800–999 and ICD10 S00-T98 codes, and an additional binary measure created for ever admitted to hospital secondary to trauma. Prescribing data were used to ascertain whether children had received at least one prescription to treat epilepsy (British National Formulary (BNF) section 4.8), diabetes (BNF section 6.1.1), attention deficit hyperactivity disorder (BNF section 4.4), and depression (tricyclic antidepressant, selective serotonin reuptake inhibitor, mirtazapine or venlafaxine), and two or more prescriptions for inhalers to treat asthma (corticosteroid in addition to long/short acting beta agonist) [21]. Neurodevelopmental disorders were defined as receipt of medication for attention deficit hyperactivity disorder or any school record of special educational need due to autistic spectrum disorder or learning disability. Mental health problems were defined as receipt of medication for depression, previous admission to a psychiatric ward/hospital or a record of special educational need attributed to mental health. The School Pupil Census was also used to identify sensory impairments, adolescent substance misuse, young carers, and children looked after by the care system.

School performance variables included the annual number of absences, annual number of exclusions for challenging/disruptive behaviour, and academic attainment across the last three years of secondary school derived from the total number of awards attained at each level of the Scottish Credit Qualifications Framework and converted into a binary variable: low/basic versus broad/general/high attainment. Absence and exclusion data were only available for years 2009, 2010 and 2012.

Maternal smoking, maternal age, parity, mode of delivery, number of admissions to hospital, ever admitted for trauma, substance misuse, looked after child, absences, exclusions and attainment were considered potentially modifiable factors, in that education, prevention, policy or practice interventions could be directed at these.

### Statistical analyses

We adhered to the statistical methods advocated for using DAGs to translate causal assumptions into statistical relationships using non-experimental data [22, 23]. Mediation models were specified for each potential mediator for which we had data. Confounders were classified as either background or intermediate confounders. Background confounders confound at least one causal pathway and do not descend from the exposure (deprivation at birth). In contrast, intermediate confounders confound a mediated pathway and are, themselves, descendants of the exposure [24]. Identifying these variables is performed by systematically applying ‘d-separation’ rules using the Dagitty software [19]. The variables included in each model are listed in S1 Table in S2 File.

The mediation models were then estimated using the user-written gformula command in Stata software. Separate models were run for each mediator. Gformula is an implementation of the G-computation procedure which permits mediation modelling in the presence of intermediate confounding, subject to certain assumptions, detailed in the ‘Sensitivity analyses’ section. Gformula estimates the total causal effect (TCE) of the exposure on the outcome and decomposes this into a natural direct effect (NDE) and a natural indirect effect (NIE) [25]. The NIE is the portion of the TCE that is transmitted through the mediator, whereas the NDE is the portion of the TCE that is transmitted through all other paths that do not involve that mediator. In the presence of intermediate confounding, the estimated effects are interpreted as randomised interventional analogues of the NDE and NIE [26]. The NDE and NIE are termed ‘natural’ because the mediator is allowed to take on the value that it would naturally take for each individual if their exposure were set to zero. Gformula also estimates the controlled direct effect (CDE), which is the unmediated effect if the mediator were fixed at the same specified value for all individuals [24]. The CDE and NDE will diverge if there is an interaction between the exposure and the mediator, and the CDE will take on different values depending on the value at which the mediator is fixed. The CDE is informative if one wishes to calculate the potential effects of a population intervention targeting the mediator: the proportion eliminated (PE) is conceptualised as the proportion of the TCE that would be removed if an intervention were implemented to set the mediator to the same value for all members of the population (e.g. an intervention that resulted in everyone becoming a non-smoker) [27]. The proportion eliminated (on the risk difference scale) is calculated as (TCE − CDE(*m*))/TCE, where *m* is the value at which the mediator is to be fixed. Since the outcome of all mediation models was binary (unemployed yes/no), gformula’s logistic function was used which returns effect sizes in the form of absolute risk differences with 95% confidence intervals (CIs) and p values. Statistical significance was defined as p<0·05. Analyses were performed on a complete case basis and missing values were not imputed, leading to different sample sizes across models.

### Sensitivity analyses

Where intermediate confounders exist, mediation analysis rests on certain assumptions regarding the inter-relationships between the variables in the model. One assumption is that there is no exposure-mediator interaction [24]. This was tested for each model using logistic regression. Where evidence of a statistically significant exposure-mediator interaction existed, we included an exposure-mediator interaction term within the relevant mediation model using gformula. However, its inclusion relied on a further assumption of no interaction between the exposure and any intermediate confounders [24]. These interactions were also tested using logistic regression. Where statistically significant, sensitivity analyses were performed to compare the gformula mediation models with and without the exposure-intermediate confounder interaction terms. Where there was evidence of variations in effect size between those models, we ran the mediation model without an exposure-intermediate confounder interaction term but stratified by each level of the intermediate confounders.

### Approvals

The study was approved by the NHS National Services Scotland Privacy Advisory Committee. A data processing agreement between Glasgow University and the Information Services Division and a data sharing agreement between Glasgow University and Scottish Exchange of Educational Data were drafted. The NHS West of Scotland Research Ethics Service confirmed that formal NHS ethics approval was not required since the study involved anonymised extracts of routinely collected data with an acceptably negligible risk of identification.

## Results

### Demographics

The study cohort comprised 217,226 former school pupils of whom 22,719 (10·5%) were NEET six months after leaving school. Study participants whose mothers were in the most deprived quintile were less likely to be Asian and their mothers were younger and more likely to have been multiparous, and to have smoked during pregnancy. They were more likely to have been delivered without obstetric assistance and have had lower gestational age and birthweight. Children born to the most deprived mothers were also more likely to have had milestone concerns, more admissions to hospital, particularly for trauma, neurodevelopmental conditions, and be treated for asthma. They were more likely to have been looked after, had more exclusions from school, and poorer attainment (Table 1).

**Table 1:** Demographics of sample by deprivation category.

		Not deprived		Deprived		Total	
		N = 155,833	71·7%	N = 61,393	28·3%	N = 217,226	
		N	%	N	%	N	%
**Demographic factors**							
Sex	Male	79,102	50·8	31,037	50·5	110,139	50·7
	Female	76,731	49·2	30,356	49·5	107,087	49·3
Ethnic group	White	149,804	96·1	59,640	97·1	209,444	97·4
	Asian	3,051	2·0·	578	0.9	3,629	1·7
	Black	64	0·0·	42	0·1	106	0·0
	Mixed	959	0·6	326	0·5	1,285	0·6
	Other	315	0·2	116	0·2	431	0·2
	Missing	2,339		0		2,339	
**Antenatal factors**							
Maternal smoking	No	97,832	75·7	25,283	50·1	123,115	68·5
	Yes	31,485	24·4	25,188	49·9	56,673	31·5
	Missing	26,516		10,922		37,438	
Maternal age (years)	<25	34,449	22·1	26,685	43·5	61,134	28·1
	25–29	56,263	36·1	19,714	32·1	75,977	35·0
	30–34	47,291	30·4	11,099	18·1	58,390	26·9
	>34	17,829	11·4	3,894	6·3	21,723	10·0
	Missing	1		1		2	
Parity	0	70,688	45·4	26,043	42·4	96,731	44·5
	1	56,307	36·1	20,411	33·3	76,718	35·3
	>1	28,813	18·5	14,924	24·3	43,737	20·1
	Missing	25		15		40	
**Perinatal factors**							
5 minute APGAR	1–3	1,129	0·7	513	0·8	1,642	0·8
	4–6	1,521	1·0·	679	1·1	2,200	1·0
	7–10	153,149	98·3	60,182	98·1	213,331	98·2
	Missing	34		19		53	
Sex, gestation-specific birthweight centile	1–3	5,741	3·7	3,633	5·9	9,374	4·3
	4–10	13,247	8·5	7,072	11·5	20,319	9·4
	11–20	18,185	11·7	8,576	14·0·	26,761	12·3
	21–80	92,973	59·8	34,583	56·4	127,556	58·7
	81–90	13,603	8·8	4,041	6·6	17,644	8·1
	91–97	8,400	5·4	2,527	4·1	10,927	5·0
	98–100	3,317	2·1	913	1·5	4,230	1·9
	Missing	367		48		415	
Gestation (weeks)	<33	1,414	0·9	733	1·2	2,147	1·0
	33	546	0·4	299	0·5	845	0·4
	34	1,103	0·7	534	0·9	1,637	0·8
	35	1,641	1·1	777	1·3	2,418	1·1
	36	3,084	2·0·	1,574	2·6	4,658	2·1
	37	7,191	4·6	3,419	5·6	10,610	4·9
	38	19,301	12·4	7,941	13·0·	27,242	12·5
	39	30,238	19·4	11,900	19·4	42,138	19·4
	40	49,637	31·9	19,250	31·4	68,887	31·7
	41	33,529	21·6	12,448	20·3	45,977	21·2
	42	7,635	4·9	2,428	4·0·	10,063	4·6
	>42	211	0·1	63	0·1	274	0·1
	Missing	303		27		330	
Mode of delivery	Unassisted	111,963	71·9	45,858	74·7	157,821	72·7
	Assisted	43,870	28·1	15,535	25·3	59,405	27·3
Congenital anomaly	No	154,478	99·1	60,815	99·1	65,315	30·1
	Yes	1,355	0·9	578	0·9	151,911	69·9
**Childhood health factors**							
Milestone concerns	No concerns	96,936	87·2	43,739	83·3	140,675	86·0
	Concerns	14,214	12·8	8,760	16·7	22,974	14·0
	Missing	44,683		8,894		53,577	
Number of admissions to hospital	0	50,564	32·4	14,751	24·0·	215,293	99·1
	>0	105,269	67·6	46,642	76·0·	1,933	0·9
Ever admitted for trauma	Yes	30,769	19·7	15,173	24·7	45,942	21·1
Epilepsy	Yes	1,237	0·8	568	0·9	1,805	0·8
Diabetes	Yes	991	0·6	340	0·6	1,331	0·6
Asthma	Yes	8,082	5·2	3,902	6·4	11,984	5·5
Neurodevelopmental concerns	Yes	5,155	3·3	3,164	5·2	8,319	3·8
Mental health problem	Yes	3,186	2·0·	1,228	2·0	4,414	2·0
Sensory impairment	Yes	867	0·6	403	0·7	1,270	0·6
**Social Factors**							
Looked after child	Yes	1,942	1·2	2,654	4·3	4,596	2·1
Young carer	Yes	76	0·1	101	0·2	177	0·1
Substance misuse	Yes	20	0·0·	55	0·1	75	0.0
**Educational factors**							
Absences	0	2,115	1·4	1,197	1·9	3,312	1·5
	>0	153,718	98·6	60,169	98·1	213,887	98·5
Exclusions	0	147,842	94·9	54,112	88·1	201,954	93·0
	>0	7,991	5·1	7,281	11·9	15,272	7·0
Attainment	Low	55,861	37·7	32,582	59·6	88,443	40·7
	High	92,393	62·3	22,126	40·4	114,519	52·7
	Missing	7,579		6,685		14,264	6·6
Leaver status	NEET	13,003	8·3	9,641	15·7	22,644	10·4
	Employed/training/education	142,830	91·7	51,752	84·3	194,582	89·6

N = Number

### Total causal effects

The point estimate for the TCE varied across the different mediation analyses due to varying sample sizes dependent on missing data within each variable. In all but one of the models, the estimated TCE was in the range 0·069 (95% CI 0·061, 0·077) to 0·076 (95% CI 0·072, 0·080) with an average of 0·073, suggesting that deprivation at birth was associated with a 7·3 percentage point increase in risk of offspring NEET six months after leaving school. This is consistent with Table 1 which demonstrated that 15·7% of deprived pupils were unemployed compared to 8·3% of their non-deprived peers (7·4% difference). The only mediation model whose TCE was not consistent with this was the attainment mediation model (TCE 0·059; 95% CI 0·040, 0·078).

### Natural direct and indirect effects

Table 2 shows the TCE, NDE and NIE for each mediation model. Of the antenatal factors, three had a statistically significant NIE. Smoking during pregnancy had a NIE of 0·016 (95% CI 0·013, 0·019), meaning that of the total effect of deprivation at birth on offspring NEET, 1·6 percentage points were mediated through smoking status. Parity had a NIE of 0·002 (95% CI 0·0003, 0·005) and younger maternal age had an NIE of 0·007 (95% CI 0·004, 0·009). Of the perinatal factors, only congenital anomalies had a statistically significant NIE of 0·003 (95% CI 0·001, 0·005). Of the childhood health mediators, admissions for trauma with an NIE of 0·003 (95% CI 0·001, 0·005) and sensory impairment with an NIE of 0·002 (95% CI 0·000, 0·004) were both statistically significant, whilst, of the social mediators only looked after child status was statistically significant, with an NIE of 0·008 (95% CI 0·006, 0·011). Of the educational factors, school absences (NIE = 0·021, 95% CI 0·018, 0·024) and exclusions (NIE = 0·003, 95% CI 0·001, 0·005) were both statistically significant. All statistically significant effects were in the expected direction; they were associated with higher rates of NEET.

**Table 2:** Mediation analysis of the effect of deprivation at birth on offspring NEET.

Mediator	Number	TCE	CI	p-value	NDE	CI	p-value	NIE	CI	p-value
**Antenatal factors**										
Smoking during pregnancy*	179,786	0·072	0·068, 0·076	<0·001	0·057	0·053, 0·061	<0·001	0·0157	0·013, 0·019	<0·001
Maternal age*	217,224	0·072	0·068, 0·076	<0·001	0·065	0·061, 0·069	<0·001	0·0067	0·004, 0·009	<0·001
Parity	217,185	0·074	0·070, 0·077	<0·001	0·071	0·068, 0·075	<0·001	0·0024	0·0003, 0·0047	0·026
**Perinatal factors**										
5 minute APGAR	179,409	0·074	0·070, 0·078	<0·001	0·074	0·070, 0·078	0·000	0·0002	-0·002, 0·003	0·863
Sex, gestation-specific birthweight centile*	179,455	0·072	0·068, 0·076	<0·001	0·075	0·071, 0·079	<0·001	-0·0023	-0·005, 0·0001	0·063
Gestation	179,527	0·075	0·071, 0·079	<0·001	0·075	0·071, 0·079	<0·001	0·0004	-0·002, 0·003	0·721
Assisted delivery	179,423	0·074	0·069, 0·078	<0·001	0·074	0·069, 0·078	<0·001	0·0001	-0·002, 0·002	0·964
Congenital anomaly	179,527	0·075	0·071, 0·079	<0·001	0·072	0·068, 0·076	<0·001	0·0029	0·001, 0·005	0·019
**Childhood health factors**										
Milestone concerns*	135,182	0·071	0·067, 0·076	<0·001	0·070	0·065, 0·074	<0·001	0·0020	-0·001, 0·004	0·285
Number of admissions to hospital*	179,457	0·076	0·072, 0·080	<0·001	0·075	0·070, 0·079	<0·001	0·0012	-0·001, 0·003	0·353
Admission for trauma	217,226	0·074	0·070, 0·077	<0·001	0·070	0·067, 0·074	<0·001	0·0033	0·001, 0·005	0·003
Epilepsy*	216,843	0·073	0·069, 0·077	<0·001	0·072	0·069, 0·076	<0·001	0·0009	-0·001, 0·003	0·420
Diabetes	216,811	0·071	0·067, 0·075	<0·001	0·072	0·068, 0·076	<0·001	-0·0010	-0·003, 0·001	0·354
Asthma*	179,529	0·076	0·072, 0·080	<0·001	0·074	0·070, 0·078	<0·001	0·0015	-0·001, 0·004	0·217
Neurodevelopmental concerns*	216,809	0·072	0·069, 0·076	<0·001	0·071	0·068, 0·075	<0·001	0·0011	-0·001, 0·003	0·345
Mental health problems	216,896	0·076	0·069, 0·082	<0·001	0·074	0·067, 0·080	<0·001	0·0020	-0·0002, 0·004	0·072
Sensory impairment	216,758	0·075	0·071, 0·079	<0·001	0·073	0·069, 0·077	<0·001	0·0022	0·00001, 0·004	0·049
**Social factors**										
Looked after child*	217,226	0·075	0·071, 0·079	<0·001	0·067	0·063, 0·071	<0·001	0·0083	0·006, 0·011	<0·001
Young carer	217,226	0·076	0·065, 0·086	<0·001	0·073	0·063, 0·084	<0·001	0·0220	-0·00001, 0·004	0·052
Substance misuse	217,226	0·072	0·051, 0·092	<0·001	0·073	0·052, 0·094	<0·001	-0·0014	-0·004, 0·0008	0·215
**Educational factors**										
Absences*	207,400	0·069	0·061, 0·077	<0·001	0·048	0·042, 0·055	<0·001	0·0207	0·018, 0·024	<0·001
Exclusions*	217,226	0·072	0·059, 0·084	<0·001	0·069	0·057, 0·081	0·000	0·0028	0·001, 0·005	0·016
Attainment*	202,962	0·059	0·040, 0·078	<0·001	0·030	0·004, 0·056	0·025	0·0296	0·013, 0·046	<0·001

TCE = Total causal effect; NDE = Natural direct effect; NIE = natural indirect effect; CI = confidence interval

*denotes models with exposure-mediator interaction term

### Controlled direct effects and proportion eliminated

As with the NDEs reported in Table 2, all p-values for the CDEs remained at <0·001. Of the antenatal factors, the PE for smoking during pregnancy was 19% (CDE when set to non-smoker 0·058; 95% CI 0·053, 0·063), the PE for parity was 27% (CDE when set to nulliparous 0·054; 95% CI 0·051, 0·058) and the PE for younger maternal age was 6% (CDE when set to age 25–29 0·068; 95% CI 0·062, 0·073). Of the perinatal factors, the PE for congenital anomalies was 1% (CDE when set to no anomaly 0·074; 95% CI 0·070, 0·078) and of childhood health mediators, the PE for admissions for trauma was 7% (CDE when set to no admissions 0·069; 95% CI 0·065, 0·072) and for sensory impairment the PE was 1% (CDE when set to no impairment 0·074; 95% CI 0·070, 0·077). Of social mediators, the PE for looked after child status was 9% (CDE when set to not looked after child 0·068; 95% CI 0·064, 0·072). Educational factors included absences, with a PE of 38% (CDE when set to no absences 0·043; 95% CI 0·037, 0·049). The CDE for exclusions was larger than the total effect (CDE when set to no exclusions 0·073; 95% CI 0·058, 0·088), possibly suggesting direct and indirect effects in opposite directions. In light of this, and the very small size of the indirect effect in Table 2, the PE is not reported here for exclusions.

### Sensitivity analyses

As indicated in Table 2, exposure-mediator interactions were found for smoking during pregnancy, maternal age, birth weight, hospital admissions, developmental milestone concerns, treated asthma and epilepsy, neurodevelopmental disorders, mental health problems, looked after child status, poor attainment, and school absences and exclusions. This indicates that any causal effect of deprivation at birth on subsequent offspring NEET that is transmitted through these mediators is non-linear, and these results will be sensitive to the prevalence of the mediator in question [28]. Consideration of interactions between the exposure and the intermediate confounders in each of these models found no notable variation in the results with and without the interaction term (see S2 Table in S2 File). Therefore Table 2 reports results from models that omitted these interactions and assumed that the conditions for causal identification were met.

## Discussion

Our study demonstrated a small but statistically significant association between deprivation at birth and offspring NEET. Offspring NEET was 7·3 percentage points higher among children born to women in the most deprived quintile. We identified some key mediators, in particular smoking during pregnancy and school absence explained 22% and 30% of the total effect respectively. Importantly, both are modifiable factors, and the estimated proportion of the total effect that could be eliminated through population interventions targeted at these mediators was 19% for smoking and 38% for school absence. Half of the total effect was mediated indirectly through lower educational attainment, which is another potential target for interventions. Therefore, our results are encouraging since they identified a small number of modifiable mediators that make substantial contributions. There are interventions known to reduce smoking during pregnancy, such as nicotine replacement therapy and counselling [29, 30]. The Scottish Government have published guidance for education authorities to promote attendance, outlining a number of strategies including parental engagement, pastoral care and providing supported learning [31].

A number of theories have been postulated as to how antenatal and early life factors can affect later health, education and employment outcomes including: altered structural brain development, a cumulative impact of multiple risk factors, family investment versus family stress mechanisms, or structural disadvantages as displayed in the social determinants of health model [4, 11, 32, 33]. The reality is that most public health problems are complex and result from multiple underlying mechanisms that are not easily studied empirically. Our study tackled this complexity through construction of a DAG depicting existing evidence and understanding of pathways that could be systematically analysed. Whilst the use of a DAG is relatively novel in this field, they are increasingly being recognised as a useful tool [14, 22, 34].

The exposure was area-based deprivation at birth whilst the outcome was offspring NEET; an individual-level indicator of deprivation. We did not have access to individual-level indicators of deprivation for mothers, such as educational level, income or employment status. It can be problematic to use maternal indicators to assess deprivation status; women’s earnings may be a poorer reflection of joint income because they earn less than their partners or are housewives [35]. Area-based measures of deprivation can be used as proxies for individual socio-economic position, although it may underestimate the true individual-level effect [36]. Measuring area-based deprivation in the offspring would not have been appropriate because six months after leaving school offspring commonly still live with their parents or in student accommodation. The outcome used, NEET, incorporates two commonly used individual-level measures of deprivation: employment status and educational level. Our study used routine data on a very large, unselected, national cohort. However, records for multiple births, children born outside of Scotland or born at home, and students attending private schools were unavailable. We estimated these to be around 3%, 12% and 5% of the national population respectively. Record linkage of 10 datasets provided data on a wide range of variables. Nonetheless, 4 variables needed to be omitted due to missing data. Data completeness was improved by combining data from multiple sources. For example, mental health problems were ascertained from school records, hospital admissions and prescriptions.

We performed sensitivity analyses with respect to exposure-mediator and exposure-confounder interactions, relevant to causal identification. There was minimal impact on the initial effect sizes, suggesting that the initial analyses were reliable and robust. The large sample size improved precision of the effect sizes estimates. We believe our DAG is a plausible, albeit simplified and not fully comprehensive, representation of the real-world relationship between deprivation at birth and subsequent offspring NEET. Our models were limited to the data available to us, therefore we acknowledge that there is likely to be residual confounding present due to the omission of several key factors, such as cognition and parenting style. Some of these residual confounders are depicted in the DAG because we suspect that they play an important role in the pathway between deprivation at birth and offspring NEET. It is also worth noting that the variables for which we had data may simply be proxy measures of other unmeasured factors that are more significant to a child’s development. Further research using additional datasets could help elicit some of these factors, and it may also be beneficial to expand data collection within existing datasets. For example, maternal and childhood adiposity are important to health but currently not well recorded in administrative datasets [37]. It is also likely that the cognitive ability of both parents and children plays an important role. This information was not available to us and should be included in future studies. A further limitation is that we analysed each mediator separately. It would be informative to work towards more comprehensive models with simultaneous estimation of multiple mediators, however methods for achieving this in the presence of intermediate confounding are not yet well-developed. A related issue is that proportions eliminated do not necessarily have a straightforward additive relationship with each other, particularly if individual mediators cause, or interact with, each other.

## Conclusions

To the best of our knowledge this is the first time a DAG has been used to understand the factors that mediate transmission of socio-economic deprivation from parents to offspring. This study illustrates the potential contribution of this novel approach in helping to disentangle such complex problems and specifically identifies key targets for intervention to obviate the perpetuation of health inequalities across generations.

## Supporting information

S1 File Excel spreadsheet detailing the structure of the directed acyclic graph. (XLSX)

S2 File (DOCX)

S1 Fig Full directed acyclic graph. (TIF)

## Abbreviations

BNF: British National Formulary
CDE: Controlled Direct Effect
CI: Confidence Interval
DAG: Directed Acyclic Graph
ICD: International Classification of Diseases
NDE: Natural Direct Effect
NEET: Not in Education, Employment or Training
NHS: National Health Service
NIE: Natural Indirect Effect
PE: Proportion Eliminated
SMR: Scottish Morbidity Record
TCE: Total Combined Effect
