# Supplementary material for: Using graphic modelling to identify modifiable mediators of the association between area-based deprivation at birth and offspring unemployment
Source: PLoS One. 2021 Mar 31;16(3):e0249258. doi: 10.1371/journal.pone.0249258 (PMC8011734; doi:10.1371/journal.pone.0249258)
Supplement: S2 File — (DOCX) [file pone.0249258.s002.docx]

**S1 Fig: Full directed acyclic graph**


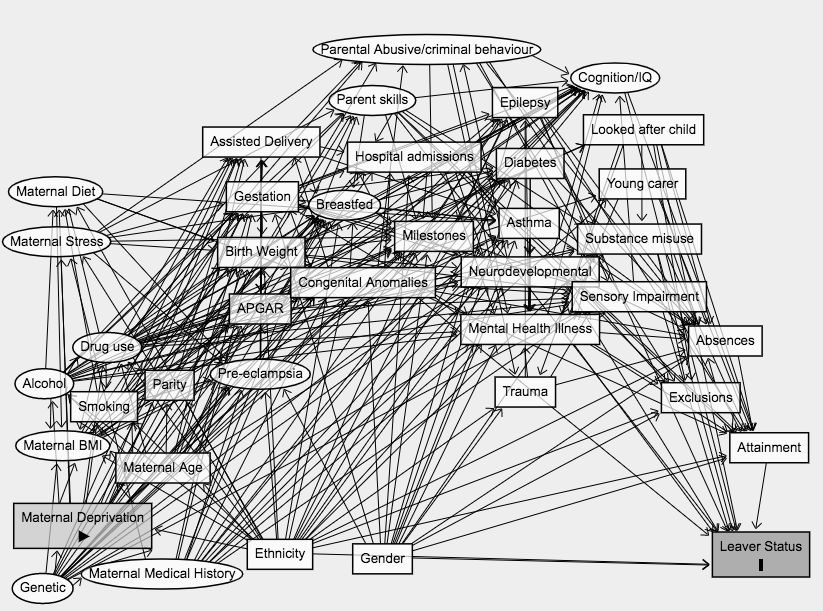


Also available at <http://dagitty.net/mW1b6V0> Square boxes indicate measured variables; round boxes indicate unmeasured variables.

**S1 Table. Structure of mediation analyses with baseline and intermediate confounders for each mediator.**

| **Category** | **Mediator** | **Baseline confounders** | **Intermediate confounders** |
| --- | --- | --- | --- |
| Antenatal | Parity | Ethnicity | Maternal age  Drug use in pregnancy (U) |
|  | Maternal age | Ethnicity |  |
|  | Smoking | Ethnicity | Maternal age  Alcohol in pregnancy (U)  Drug use in pregnancy (U) |
| Perinatal | APGAR score | Ethnicity  Maternal medical history (U)  Genetic and family history (U) | Birthweight  Congenital anomaly  Drug use in pregnancy (U)  Gestation  Maternal adiposity (U)  Maternal age  Mode of delivery  Pre-eclampsia (U)  Smoking |
|  | Gestational age | Ethnicity  Sex  Maternal medical history (U)  Genetic and family history (U) | Alcohol (U)  Congenital anomalies  Drug use in pregnancy (U)  Maternal adiposity (U)  Maternal age  Maternal diet (U)  Maternal stress (U)  Pre-eclampsia (U)  Smoking (U) |
|  | Assisted delivery | Ethnicity  Maternal medical history (U)  Genetic and family history (U) | Birthweight  Congenital anomalies  Drug use in pregnancy (U)  Gestation  Maternal adiposity (U)  Maternal age  Parity  Pre-eclampsia (U)  Smoking |
|  | Congenital anomaly | Ethnicity  Sex  Maternal medical history (U)  Genetic and family history (U) | Alcohol use in pregnancy (U)  Drug use in pregnancy (U)  Gestation  Maternal adiposity (U)  Maternal age  Maternal diet (U)  Maternal stress (U)  Pre-eclampsia (U)  Smoking |
|  | Birthweight | Ethnicity  Maternal medical history (U)  Genetic and family history (U) | Alcohol in pregnancy (U)  Congenital anomaly  Drug use in pregnancy (U)  Gestation  Maternal adiposity (U) |
| Early Life | Milestone concern | Ethnicity  Sex  Maternal medical history (U)  Genetic and family history (U) | APGAR  Abusive/criminal behaviour (U)  Alcohol in pregnancy (U)  Asthma  Birthweight  Congenital anomalies  Diabetes  Drug use in pregnancy (U)  Epilepsy  Breastfed (U)  Gestation  Hospital admissions  Neurodevelopmental  Parental skills (U)  Sensory impairment  Smoking |
| Childhood Health | Trauma admission | Ethnicity  Sex  Maternal medical history (U)  Genetic and family history (U) | Abusive/criminal behaviour (U)  Looked after child |
|  | Diabetes | Ethnicity  Sex  Maternal medical history (U)  Genetic and family history (U) | Birthweight  Breastfed  Gestation  Maternal adiposity |
|  | Epilepsy | Ethnicity  Sex  Maternal medical history (U)  Genetic and family history (U) | APGAR  Congenital anomalies  Gestation  Trauma |
|  | Number of hospital admissions | Ethnicity  Sex  Maternal medical history (U)  Genetic and family history (U) | Birthweight  Congenital anomalies  Breastfeeding (U)  Gestation  Assisted delivery  Parental Skills (U)  Smoking |
|  | Asthma | Ethnicity  Sex  Maternal medical history (U)  Genetic and family history (U) | Breastfed  Gestation  Hospital admissions  Maternal adiposity (U)  Maternal diet (U)  Assisted delivery  Smoking |
|  | Neurodevelopmental concern | Ethnicity  Sex  Maternal medical history (U)  Genetic and family history (U) | Alcohol use in pregnancy (U)  Asthma  Birthweight  Congenital anomalies  Diabetes  Drug use in pregnancy (U)  Breasted (U)  Gestation  Hospital admissions  Maternal age |
|  | Mental health problem | Ethnicity  Sex  Maternal medical history (U)  Genetic and family history (U) | Abusive/criminal behaviour (U)  Alcohol use in pregnancy (U)  Asthma  Congenital anomalies  Diabetes  Drug use in pregnancy (U)  Epilepsy  Gestation  Hospital admissions  Looked after child  Neurodevelopmental  Sensory impairment  Trauma  Young Carer |
| School engagement | Sensory impairment | Ethnicity  Maternal medical history (U)  Genetic and family history (U) | APGAR  Abusive/criminal behaviour (U)  Alcohol use in pregnancy (U)  Birthweight  Congenital anomalies  Drug use in pregnancy (U)  Epilepsy  Gestation  Hospital admissions  Neurodevelopmental  Trauma |
|  | Young carer | Ethnicity  Maternal medical history (U) | Drug use in pregnancy |
|  | Substance misuse | Ethnicity  Sex  Maternal medical history (U)  Genetic and family history (U) | Abusive/criminal behaviour (U)  Alcohol use in pregnancy (U)  Drug use in pregnancy (U)  Looked after child  Mental health issues  Parental skills (U) |
|  | Looked after child | Ethnicity | Alcohol use in pregnancy (U)  Drug use in pregnancy (U) |
| Education | Absences | Ethnicity  Sex  Maternal medical history (U)  Genetic and family history (U) | Cognition/IQ (U)  Congenital anomaly  Epilepsy  Exclusions  Looked after child  Mental health problem  Neurodevelopmental  Parental skills (U)  Sensory impairment  Young carer |
|  | Exclusions | Ethnicity  Sex  Maternal medical history (U)  Genetic and family history (U) | Abusive/criminal behaviour (U)  Looked after child  Mental health problem  Neurodevelopmental  Parental skills (U)  Substance misuse |
|  | Academic attainment | Ethnicity  Sex  Maternal medical history (U)  Genetic and family history (U) | Cognition/IQ (U)  Congenital anomaly  Epilepsy  Looked after child  Mental health problem  Neurodevelopmental  Parental skills (U)  Sensory impairment  Substance misuse |

(U) denotes an unmeasured variable

**S2 Table. Gformula mediation results with inclusion of mediator-Intermediate confounder interaction terms.**

| **Mediator (N)** | **TCE** | **CI** | **p-value** | **NDE** | **CI** | **p-value** | **NIE** | **CI** | **p-value** |
| --- | --- | --- | --- | --- | --- | --- | --- | --- | --- |
| Smoking (179,786) | 0·073 | 0·069, 0·077 | <0·001 | 0·056 | 0·052, 0·061 | <0·001 | 0·016 | 0·013, 0·019 | <0·001 |
| Birthweight (179,455) | 0·074 | 0·070, 0·078 | <0·001 | 0·074 | 0·070, 0·079 | <0·001 | -0·001 | -0·003, 0·002 | 0·493 |
| Admissions (179,457) | 0·076 | 0·072, 0·081 | <0·001 | 0·075 | 0·071, 0·079 | <0·001 | 0·001 | -0·001, 0·004 | 0·221 |
| Milestone concern (135,182) | 0·071 | 0·067, 0·076 | <0·001 | 0·070 | 0·065, 0·074 | <0·001 | 0·002 | -0·001, 0·005 | 0·239 |
| Asthma (179,529) | 0·076 | 0·072, 0·080 | <0·001 | 0·074 | 0·070, 0·078 | <0·001 | 0·002 | -0·0003, 0·005 | 0·091 |
| Neurodevelopmental disorder (216,809) | 0·073 | 0·069, 0·076 | <0·001 | 0·072 | 0·068, 0·075 | <0·001 | 0·001 | -0·001, 0·004 | 0·225 |
| Mental health problem (216,896) | 0·076 | 0·069, 0·083 | <0·001 | 0·074 | 0·067, 0·081 | <0·001 | 0·002 | -0·0004, 0·004 | 0·100 |
| Absences (207,400) | 0·069 | 0·061, 0·077 | <0·001 | 0·049 | 0·042, 0·055 | <0·001 | 0·021 | 0·018, 0·024 | <0·001 |
| Exclusions (217,226) | 0·071 | 0·059, 0·084 | <0·001 | 0·069 | 0·057, 0·081 | <0·001 | 0·003 | 0·0004, 0·005 | 0·021 |

**Definition of Scottish Index of Multiple Deprivations.**

Referenced from: Gov.scot. 2021. Scottish Index Of Multiple Deprivation 2020 - Gov.Scot. [online] Available at: <https://www.gov.scot/collections/scottish-index-of-multiple-deprivation-2020/> [Accessed 25 January 2021].

The Scottish Index of Multiple Deprivations (SIMD) is a area-based, relative, composite measure of deprivation across 6,976 data zones. Each zone is ranked from most deprived (ranked 1) to least deprived (ranked 6,976).

The 2020 SIMD uses 37 indicators from 7 domains to assess deprivation status. Those domains are Income, Employment, Education, Health, Access to services, Crime and Housing.

**S1 File: Excel spreadsheet detailing the structure of the directed acyclic graph**

**References for the directed acyclic graph**

1. de Graaf JP, Steegers EA, Bonsel GJ. Inequalities in perinatal and maternal health. *Curr Opin Obstet Gynecol*. 2013;25(2):98-108. doi:10.1097/GCO.0b013e32835ec9b0
2. Beauregard JL, Drews-Botsch C, Sales JM, Flanders WD, Kramer MR. Preterm Birth, Poverty, and Cognitive Development. *Pediatrics*. 2018;141(1):e20170509. doi:10.1542/peds.2017-0509
3. Gray, R., Bonellie, S., Chalmers, J., Greer, I., Jarvis, S. and Williams, C. Social inequalities in preterm birth in Scotland 1980–2003: findings from an area‐based measure of deprivation. *BJOG: An International Journal of Obstetrics & Gynaecology*, 2008;115: 82-90. <https://doi.org/10.1111/j.1471-0528.2007.01582.x>
4. Fairley L, Leyland AH. Social class inequalities in perinatal outcomes: Scotland 1980-2000. *J Epidemiol Community Health*. 2006;60(1):31-36. doi:10.1136/jech.2005.038380
5. Martin RP, Dombrowski SC, Mullis C, Wisenbaker J, Huttunen MO. Smoking during pregnancy: association with childhood temperament, behavior, and academic performance. *J Pediatr Psychol*. 2006;31(5):490-500. doi:10.1093/jpepsy/jsj041
6. McCall SJ, Bhattacharya S, Okpo E, Macfarlane GJ. Evaluating the social determinants of teenage pregnancy: a temporal analysis using a UK obstetric database from 1950 to 2010. *J Epidemiol Community Health*. 2015;69(1):49-54. doi:10.1136/jech-2014-204214
7. Aizer A, Currie J. The intergenerational transmission of inequality: maternal disadvantage and health at birth. *Science*. 2014;344(6186):856-861. doi:10.1126/science.1251872
8. Kramer MS, Séguin L, Lydon J, Goulet L. Socio-economic disparities in pregnancy outcome: why do the poor fare so poorly?. *Paediatr Perinat Epidemiol*. 2000;14(3):194-210. doi:10.1046/j.1365-3016.2000.00266.x
9. Spong CY, Mercer BM, D'alton M, Kilpatrick S, Blackwell S, Saade G. Timing of indicated late-preterm and early-term birth. *Obstet Gynecol*. 2011;118(2 Pt 1):323-333. doi:10.1097/AOG.0b013e3182255999
10. Emerson E. Deprivation, ethnicity and the prevalence of intellectual and developmental disabilities. *J Epidemiol Community Health*. 2012;66(3):218-224. doi:10.1136/jech.2010.111773
11. Emerson E, Hatton C. Contribution of socioeconomic position to health inequalities of British children and adolescents with intellectual disabilities. *Am J Ment Retard*. 2007;112(2):140-150. doi:10.1352/0895-8017(2007)112[140:COSPTH]2.0.CO;2
12. Emerson E, Hatton C. Mental health of children and adolescents with intellectual disabilities in Britain. *Br J Psychiatry*. 2007;191:493-499. doi:10.1192/bjp.bp.107.038729
13. Newschaffer CJ, Croen LA, Daniels J, et al. The epidemiology of autism spectrum disorders. *Annu Rev Public Health*. 2007;28:235-258. doi:10.1146/annurev.publhealth.28.021406.144007
14. Anders, Y., Sammons, P., Taggart, B., Sylva, K., Melhuish, E. and Siraj‐Blatchford, I. The influence of child, family, home factors and pre‐school education on the identification of special educational needs at age 10. *British Educational Research Journal*, 2011;37: 421-441. <https://doi.org/10.1080/01411921003725338>
15. MacKay DF, Smith GC, Dobbie R, Pell JP. Gestational age at delivery and special educational need: retrospective cohort study of 407,503 schoolchildren. *PLoS Med*. 2010;7(6):e1000289. Published 2010 Jun 8. doi:10.1371/journal.pmed.1000289
16. Mackay DF, Smith GC, Dobbie R, Cooper SA, Pell JP. Obstetric factors and different causes of special educational need: retrospective cohort study of 407,503 schoolchildren. *BJOG*. 2013;120(3):297-308. doi:10.1111/1471-0528.12071
17. Tweed EJ, Mackay DF, Nelson SM, Cooper SA, Pell JP. Five-minute Apgar score and educational outcomes: retrospective cohort study of 751,369 children. *Arch Dis Child Fetal Neonatal Ed*. 2016;101(2):F121-F126. doi:10.1136/archdischild-2015-308483
18. Iliodromiti S, Mackay DF, Smith GC, Pell JP, Nelson SM. Apgar score and the risk of cause-specific infant mortality: a population-based cohort study. *Lancet*. 2014;384(9956):1749-1755. doi:10.1016/S0140-6736(14)61135-1
19. Vrijheid M, Dolk H, Stone D, Abramsky L, Alberman E, Scott JE. Socioeconomic inequalities in risk of congenital anomaly. *Arch Dis Child*. 2000;82(5):349-352. doi:10.1136/adc.82.5.349
20. Playford, C.J., Dibben, C. & Williamson, L. Socioeconomic disadvantage, fetal environment and child development: linked Scottish administrative records based study. *Int J Equity Health* 2017;16, (203) <https://doi.org/10.1186/s12939-017-0698-4>
21. Spencer N, Strazdins L. Socioeconomic disadvantage and onset of childhood chronic disabling conditions: a cohort study. *Arch Dis Child*. 2015;100(4):317-322. doi:10.1136/archdischild-2013-305634
22. Duncan GJ, Magnuson K, Votruba-Drzal E. Moving Beyond Correlations in Assessing the Consequences of Poverty. *Annu Rev Psychol*. 2017;68:413-434. doi:10.1146/annurev-psych-010416-044224
23. Stewart CH, Dundas R, Leyland AH. The Scottish school leavers cohort: linkage of education data to routinely collected records for mortality, hospital discharge and offspring birth characteristics. *BMJ Open*. 2017;7(7):e015027. Published 2017 Jul 10. doi:10.1136/bmjopen-2016-015027
24. Howe LD, Lawlor DA, Propper C. Trajectories of socioeconomic inequalities in health, behaviours and academic achievement across childhood and adolescence. *J Epidemiol Community Health*. 2013;67(4):358-364. doi:10.1136/jech-2012-201892
25. Morris T, Dorling D, Davey Smith G. How well can we predict educational outcomes? Examining the roles of cognitive ability and social position in educational attainment. *Contemp Soc Sci*. 2016;11(2-3):154-168. doi:10.1080/21582041.2016.1138502
26. Gov.scot. 2020. *The Life Chances Of Young People In Scotland: Report To The First Minister - Gov.Scot*. [online] Available at: <https://www.gov.scot/publications/independent-advisor-poverty-inequality-life-chances-young-people-scotland-report/pages/8/> [Accessed 2 December 2020].
27. Abrahams, D. (Ed.). (2016). *Inquiry: Child Poverty and Health*. London, UK: All Party Parliamentary Group on Health in All Policies.
28. McAndrew, F., 2012. *Infant Feeding Survey 2010*. 1st ed. London: Health and Social Care Information Centre.
29. Odd D, Lewis G, Gunnell D, Rasmussen F. Risk of low Apgar scores and socioeconomic status over a 30-year period. *J Matern Fetal Neonatal Med*. 2014;27(6):603-607. doi:10.3109/14767058.2013.833903
30. Odd DE, Lewis G, Whitelaw A, Gunnell D. Resuscitation at birth and cognition at 8 years of age: a cohort study. *Lancet*. 2009;373(9675):1615-1622. doi:10.1016/S0140-6736(09)60244-0
31. Odd DE, Rasmussen F, Gunnell D, Lewis G, Whitelaw A. A cohort study of low Apgar scores and cognitive outcomes. *Arch Dis Child Fetal Neonatal Ed*. 2008;93(2):F115-F120. doi:10.1136/adc.2007.123745
32. Rogers JM. Tobacco and pregnancy. *Reprod Toxicol*. 2009;28(2):152-160. doi:10.1016/j.reprotox.2009.03.012
33. Subbarao P, Mandhane PJ, Sears MR. Asthma: epidemiology, etiology and risk factors. *CMAJ*. 2009;181(9):E181-E190. doi:10.1503/cmaj.080612
34. Been JV, Lugtenberg MJ, Smets E, et al. Preterm birth and childhood wheezing disorders: a systematic review and meta-analysis. *PLoS Med*. 2014;11(1):e1001596. Published 2014 Jan 28. doi:10.1371/journal.pmed.1001596
35. Jaakkola JJ, Ahmed P, Ieromnimon A, et al. Preterm delivery and asthma: a systematic review and meta-analysis. *J Allergy Clin Immunol*. 2006;118(4):823-830. doi:10.1016/j.jaci.2006.06.043
36. Li S, Zhang M, Tian H, Liu Z, Yin X, Xi B. Preterm birth and risk of type 1 and type 2 diabetes: systematic review and meta-analysis. *Obes Rev*. 2014;15(10):804-811. doi:10.1111/obr.12214
37. Hirvonen M, Ojala R, Korhonen P, et al. The incidence and risk factors of epilepsy in children born preterm: A nationwide register study. *Epilepsy Res*. 2017;138:32-38. doi:10.1016/j.eplepsyres.2017.10.005
38. Whitehead E, Dodds L, Joseph KS, et al. Relation of pregnancy and neonatal factors to subsequent development of childhood epilepsy: a population-based cohort study. *Pediatrics*. 2006;117(4):1298-1306. doi:10.1542/peds.2005-1660
39. Rantakallio P, von Wendt L. Prognosis for low-birthweight infants up to the age of 14: a population study. *Dev Med Child Neurol*. 1985;27(5):655-663. doi:10.1111/j.1469-8749.1985.tb14138.x
40. Golding J. Determinants of child health and development: the contribution of ALSPAC--a personal view of the birth cohort study. *Arch Dis Child*. 2010;95(5):319-322. doi:10.1136/adc.2009.178954
41. Spencer, N. The social determinants of child health. *Paediatrics and Child Health*, 2018;28(3), pp.138-143.
42. State of Child Health Report 2017. Rcpch.ac.uk. https://www.rcpch.ac.uk/sites/default/files/2018-05/state_of_child_health_2017report_updated_29.05.18.pdf. Published 2017. Accessed December 2, 2020.
43. Turney K, Lee H, Mehta N. The social determinants of child health. *Soc Sci Med*. 2013;95:1-5. doi:10.1016/j.socscimed.2013.07.015
44. Kotch J. *Maternal And Child Health*. Sudbury, Mass.: Jones & Bartlett Learning; 2013.
45. Growing up in the UK: ensuring a healthy future for our children. The British Medical Association is the trade union and professional body for doctors in the UK. https://www.bma.org.uk/what-we-do/population-health/child-health/growing-up-in-the-uk-ensuring-a-healthy-future-for-our-children. Published 2013. Accessed December 2, 2020.
46. Shah PS; Knowledge Synthesis Group on Determinants of LBW/PT births. Parity and low birth weight and preterm birth: a systematic review and meta-analyses. *Acta Obstet Gynecol Scand*. 2010;89(7):862-875. doi:10.3109/00016349.2010.486827
47. Palmer M, Silverwood RJ. Socioeconomic disadvantage and childhood growth: A review of the literature focusing on the mediatory roles of birth weight, maternal age and parity. NCRM Working Paper. 2014. <http://eprints.ncrm.ac.uk/3716/>.
48. Mol B, Roberts C, Thangaratinam S, Magee L, de Groot C, Hofmeyr G. Pre-eclampsia. *The Lancet*. 2016;387(10022):999-1011. doi:10.1016/s0140-6736(15)00070-7
49. Walsh RA, Redman S, Brinsmead MW, Fryer JL. Predictors of smoking in pregnancy and attitudes and knowledge of risks of pregnant smokers. *Drug Alcohol Rev*. 1997;16(1):41-67. doi:10.1080/09595239700186321
50. Lean SC, Derricott H, Jones RL, Heazell AEP. Advanced maternal age and adverse pregnancy outcomes: A systematic review and meta-analysis. *PLoS One*. 2017;12(10):e0186287. Published 2017 Oct 17. doi:10.1371/journal.pone.0186287
51. de Vienne CM, Creveuil C, Dreyfus M. Does young maternal age increase the risk of adverse obstetric, fetal and neonatal outcomes: a cohort study. *Eur J Obstet Gynecol Reprod Biol*. 2009;147(2):151-156. doi:10.1016/j.ejogrb.2009.08.006
52. Malabarey OT, Balayla J, Klam SL, Shrim A, Abenhaim HA. Pregnancies in young adolescent mothers: a population-based study on 37 million births. *J Pediatr Adolesc Gynecol*. 2012;25(2):98-102. doi:10.1016/j.jpag.2011.09.004
53. Colombo L, Crippa BL, Consonni D, et al. Breastfeeding Determinants in Healthy Term Newborns. *Nutrients*. 2018;10(1):48. Published 2018 Jan 5. doi:10.3390/nu10010048
54. Births in Scottish Hospitals. Isdscotland.org. https://www.isdscotland.org/Health-Topics/Maternity-and-Births/Publications/2017-11-28/2017-11-28-Births-Report.pdf. Published 2017. Accessed December 2, 2020.
55. Bhuvaneswar CG, Chang G, Epstein LA, Stern TA. Alcohol use during pregnancy: prevalence and impact. *Prim Care Companion J Clin Psychiatry*. 2007;9(6):455-460. doi:10.4088/pcc.v09n0608
56. Straube S, Voigt M, Jorch G, Hallier E, Briese V, Borchardt U. Investigation of the association of Apgar score with maternal socio-economic and biological factors: an analysis of German perinatal statistics. *Arch Gynecol Obstet*. 2010;282(2):135-141. doi:10.1007/s00404-009-1217-7
57. Hemminki E, Malin M, Rahkonen O. Mother's social class and perinatal problems in a low-problem area. *Int J Epidemiol*. 1990;19(4):983-990. doi:10.1093/ije/19.4.983
58. Netto, G., Sosenko, F. & Bramley, G. Poverty And Ethnicity In Scotland. Review Of The Literature And Datasets. *Joseph Rowntree Foundation* (Ed.). 2011. York.
59. Samuel Lurie, Shay Ribenzaft, Mona Boaz, Abraham Golan & Oscar Sadan. The effect of cigarette smoking during pregnancy on mode of delivery in uncomplicated term singleton pregnancies, *The Journal of Maternal-Fetal & Neonatal Medicine.* 2014;27(8), 812-815, DOI: [10.3109/14767058.2013.842551](https://doi.org/10.3109/14767058.2013.842551)
60. Räisänen S, Gissler M, Kramer MR, Heinonen S. Influence of delivery characteristics and socioeconomic status on giving birth by caesarean section - a cross sectional study during 2000-2010 in Finland. *BMC Pregnancy Childbirth*. 2014;14:120. Published 2014 Mar 31. doi:10.1186/1471-2393-14-120
61. Silva LM, Coolman M, Steegers EA, et al. Low socioeconomic status is a risk factor for preeclampsia: the Generation R Study. *J Hypertens*. 2008;26(6):1200-1208. doi:10.1097/HJH.0b013e3282fcc36e
62. Amir LH, Donath SM. Does maternal smoking have a negative physiological effect on breastfeeding? The epidemiological evidence. *Birth*. 2002;29(2):112-123. doi:10.1046/j.1523-536x.2002.00152.x
63. Conde-Agudelo A, Althabe F, Belizán JM, Kafury-Goeta AC. Cigarette smoking during pregnancy and risk of preeclampsia: a systematic review. *Am J Obstet Gynecol*. 1999;181(4):1026-1035. doi:10.1016/s0002-9378(99)70341-8
64. Hackshaw A, Rodeck C, Boniface S. Maternal smoking in pregnancy and birth defects: a systematic review based on 173 687 malformed cases and 11.7 million controls. *Hum Reprod Update*. 2011;17(5):589-604. doi:10.1093/humupd/dmr022
65. Amir, L.H., Donath, S. A systematic review of maternal obesity and breastfeeding intention, initiation and duration. *BMC Pregnancy Childbirth*. 2007;7, (9). <https://doi.org/10.1186/1471-2393-7-9>
66. Behrman RE, Butler AS, Institute of Medicine (US) Committee on Understanding Premature Birth and Assuring Healthy Outcomes, eds. *Preterm Birth: Causes, Consequences, and Prevention*. Washington (DC): National Academies Press (US); 2007.
67. Zeitlin J, Saurel-Cubizolles MJ, De Mouzon J, et al. Fetal sex and preterm birth: are males at greater risk?. *Hum Reprod*. 2002;17(10):2762-2768. doi:10.1093/humrep/17.10.2762
68. Eiríksdóttir VH, Ásgeirsdóttir TL, Bjarnadóttir RI, Kaestner R, Cnattingius S, et al. Low Birth Weight, Small for Gestational Age and Preterm Births before and after the Economic Collapse in Iceland: A Population Based Cohort Study. *PLOS ONE* 2013;8(12): e80499. <https://doi.org/10.1371/journal.pone.0080499>
69. Bale J, Stoll B, Lucas A. *Reducing Birth Defects*. Washington, DC: National Academies Press; 2003.
70. Gu H, Wang L, Liu L, et al. A gradient relationship between low birth weight and IQ: A meta-analysis. *Sci Rep*. 2017;7(1):18035. Published 2017 Dec 21. doi:10.1038/s41598-017-18234-9
71. Levine SZ. Low birth-weight and risk for major depression: a community-based longitudinal study. *Psychiatry Res*. 2014;215(3):618-623. doi:10.1016/j.psychres.2014.01.008
72. Loret de Mola C, de França GV, Quevedo Lde A, Horta BL. Low birth weight, preterm birth and small for gestational age association with adult depression: systematic review and meta-analysis. *Br J Psychiatry*. 2014;205(5):340-347. doi:10.1192/bjp.bp.113.139014
73. Xu XF, Li YJ, Sheng YJ, Liu JL, Tang LF, Chen ZM. Effect of low birth weight on childhood asthma: a meta-analysis. *BMC Pediatr*. 2014;14:275. Published 2014 Oct 23. doi:10.1186/1471-2431-14-275
74. Stene LC, Magnus P, Lie RT, Søvik O, Joner G; Norwegian childhood Diabetes Study Group. Birth weight and childhood onset type 1 diabetes: population based cohort study. *BMJ*. 2001;322(7291):889-892. doi:10.1136/bmj.322.7291.889
75. Nagy E, Orvos H, Bakki J, Pal A. Sex-differences in Apgar scores for full-term neonates. *Acta Paediatr*. 2009;98(5):898-900. doi:10.1111/j.1651-2227.2009.01238.x
76. Black M, Bhattacharya S, Philip S, Norman JE, McLernon DJ. Planned Cesarean Delivery at Term and Adverse Outcomes in Childhood Health. *JAMA*. 2015;314(21):2271-2279. doi:10.1001/jama.2015.16176
77. Eogan MA, Geary MP, O'Connell MP, Keane DP. Effect of fetal sex on labour and delivery: retrospective review. *BMJ*. 2003;326(7381):137. doi:10.1136/bmj.326.7381.137
78. Bokslag A, van Weissenbruch M, Mol BW, de Groot CJ. Preeclampsia; short and long-term consequences for mother and neonate. *Early Hum Dev*. 2016;102:47-50. doi:10.1016/j.earlhumdev.2016.09.007
79. Jaskolka D, Retnakaran R, Zinman B, Kramer CK. Fetal sex and maternal risk of pre-eclampsia/eclampsia: a systematic review and meta-analysis. *BJOG*. 2017;124(4):553-560. doi:10.1111/1471-0528.14163
80. Fleming M. *Using Scotland-Wide Record Linkage To Investigate The Educational And Health Outcomes Of Children Treated For Chronic Conditions*. 1st ed. Glasgow: University of Glasgow; 2017.
81. Piccini P, Montagnani C, de Martino M. Gender disparity in pediatrics: a review of the current literature. *Ital J Pediatr*. 2018;44(1):1. Published 2018 Jan 2. doi:10.1186/s13052-017-0437-x
82. June M. Reinisch PhD, Leonard A. Rosenblum PhD, Donald B. Rubin PhD & M. Fini Schulsinger MD. Sex Differences in Developmental Milestones During the First Year of Life, Journal of Psychology & Human Sexuality, 1991;4(2), 19-36, DOI: [10.1300/J056v04n02_03](https://doi.org/10.1300/J056v04n02_03)
83. Tennant PW, Samarasekera SD, Pless-Mulloli T, Rankin J. Sex differences in the prevalence of congenital anomalies: a population-based study. *Birth Defects Res A Clin Mol Teratol*. 2011;91(10):894-901. doi:10.1002/bdra.22846
84. Cremers CW, van Rijn PM, Huygen PL. The sex-ratio in childhood deafness, an analysis of the male predominance. *Int J Pediatr Otorhinolaryngol*. 1994;30(2):105-110. doi:10.1016/0165-5876(94)90192-9
85. IOM (Institute of Medicine), 2012. [*Epilepsy Across the Spectrum: Promoting health and understanding.*](http://nationalacademies.org/HMD/Reports/2012/Epilepsy-Across-the-Spectrum.aspx) Washington, DC: The National Academies Press.
86. Panico L, Bartley M, Marmot M, Nazroo JY, Sacker A, Kelly YJ. Ethnic variation in childhood asthma and wheezing illnesses: findings from the Millennium Cohort Study. *Int J Epidemiol*. 2007;36(5):1093-1102. doi:10.1093/ije/dym089
87. Dwivedi KN, Banhatti RG. Attention deficit/hyperactivity disorder and ethnicity. *Archives of Disease in Childhood*2005;90**:**i10-i12.
88. Bhattachayya G, Ison L, Blair M (2003), Minority Ethnic Attainment and Participation in Education and Training: The Evidence, Research Topic paper RTP01-03, Department for Education and Skills
89. Santangeli L, Sattar N, Huda SS. Impact of maternal obesity on perinatal and childhood outcomes. *Best Pract Res Clin Obstet Gynaecol*. 2015;29(3):438-448. doi:10.1016/j.bpobgyn.2014.10.009
90. Rusconi F, Popovic M. Maternal obesity and childhood wheezing and asthma. *Paediatr Respir Rev*. 2017;22:66-71. doi:10.1016/j.prrv.2016.08.009
91. Lambert BL, Bauer CR. Developmental and behavioral consequences of prenatal cocaine exposure: a review. *J Perinatol*. 2012;32(11):819-828. doi:10.1038/jp.2012.90
92. Marquis GS. Breastfeeding and Its Impact on Child Psychosocial and Emotional Development: Comments on Woodward and Liberty, Greiner, Pérez-Escamilla, and Lawrence. In: Tremblay RE, Boivin M, Peters RDeV, eds. *Encyclopedia on Early Childhood Development* [online]. <http://www.child-encyclopedia.com/breastfeeding/according-experts/breastfeeding-and-its-impact-child-psychosocial-and-emotional>. Updated March 2008. Accessed December 2, 2020.
93. Cardwell CR, Stene LC, Ludvigsson J, et al. Breast-feeding and childhood-onset type 1 diabetes: a pooled analysis of individual participant data from 43 observational studies. *Diabetes Care*. 2012;35(11):2215-2225. doi:10.2337/dc12-0438
94. Murray, G.K., Jones, P.B., Kuh, D. and Richards, M. Infant developmental milestones and subsequent cognitive function. Ann Neurol., 2007;62: 128-136. <https://doi.org/10.1002/ana.21120>
95. Propper, C., & Rigg, J. (2006). Understanding socio-economic inequalities in childhood respiratory health. London: Centre for Analysis of Social Exclusion
96. Meschke LL, Holl J, Messelt S. Older not wiser: risk of prenatal alcohol use by maternal age. *Matern Child Health J*. 2013;17(1):147-155. doi:10.1007/s10995-012-0953-7
97. Stothard KJ, Tennant PW, Bell R, Rankin J. Maternal overweight and obesity and the risk of congenital anomalies: a systematic review and meta-analysis. *JAMA*. 2009;301(6):636-650. doi:10.1001/jama.2009.113
98. Goldenberg RL, Culhane JF, Iams JD, Romero R. Epidemiology and causes of preterm birth. *Lancet*. 2008;371(9606):75-84. doi:10.1016/S0140-6736(08)60074-4
99. Mattson SN, Crocker N, Nguyen TT. Fetal alcohol spectrum disorders: neuropsychological and behavioral features. *Neuropsychol Rev*. 2011;21(2):81-101. doi:10.1007/s11065-011-9167-9
100. Young SL, Vosper HJ, Phillips SA. Cocaine: its effects on maternal and child health. *Pharmacotherapy.* 1992;12:2-17.
101. Ji J, Hemminki K, Sundquist J, Sundquist K. Ethnic differences in incidence of type 1 diabetes among second-generation immigrants and adoptees from abroad. *J Clin Endocrinol Metab*. 2010;95(2):847-850. doi:10.1210/jc.2009-1818
102. Jones E, Gutman L, Platt L. Family Stressors and Children's Outcomes. Bettercarenetwork.org. https://bettercarenetwork.org/sites/default/files/attachments/Family%20Stressors%20and%20Children%27s%20Outcomes.pdf. Published 2013. Accessed December 3, 2020.
103. Pallavi Amitava Banerjee | Stephen Lamb (Reviewing Editor). A systematic review of factors linked to poor academic performance of disadvantaged students in science and maths in schools, *Cogent Education*, 2016;3:1, DOI: [10.1080/2331186X.2016.1178441](https://doi.org/10.1080/2331186X.2016.1178441)
104. Taylor RM, Fealy SM, Bisquera A, et al. Effects of Nutritional Interventions during Pregnancy on Infant and Child Cognitive Outcomes: A Systematic Review and Meta-Analysis. *Nutrients*. 2017;9(11):1265. Published 2017 Nov 20. doi:10.3390/nu9111265
105. Srám RJ, Binková B, Dejmek J, Bobak M. Ambient air pollution and pregnancy outcomes: a review of the literature. *Environ Health Perspect*. 2005;113(4):375-382. doi:10.1289/ehp.6362
106. Vrijheid M, Casas M, Gascon M, Valvi D, Nieuwenhuijsen M. Environmental pollutants and child health-A review of recent concerns. *Int J Hyg Environ Health*. 2016;219(4-5):331-342. doi:10.1016/j.ijheh.2016.05.001
107. Hinman SK, Smith KB, Quillen DM, Smith MS. Exercise in Pregnancy: A Clinical Review. *Sports Health*. 2015;7(6):527-531. doi:10.1177/1941738115599358
108. Scharte M, Bolte G. Kinder alleinerziehender Frauen in Deutschland: Gesundheitsrisiken und Umweltbelastungen [Children of single mothers: health risks and environmental stress]. *Gesundheitswesen*. 2012;74(3):123-131. doi:10.1055/s-0030-1270507
109. National Research Council. 1993. Understanding Child Abuse and Neglect. Washington, DC: The National Academies Press. <https://doi.org/10.17226/2117>.
110. Grinde, B., Tambs, K. Effect of household size on mental problems in children: results from the Norwegian Mother and Child Cohort study. *BMC Psychol* 2016;4:31. <https://doi.org/10.1186/s40359-016-0136-1>
111. Muenchhoff M, Goulder PJ. Sex differences in pediatric infectious diseases. *J Infect Dis*. 2014;209 Suppl 3(Suppl 3):S120-S126. doi:10.1093/infdis/jiu232
112. Köhler-Forsberg O, Sørensen HJ, Nordentoft M, McGrath JJ, Benros ME, Petersen L. Childhood Infections and Subsequent School Achievement Among 598,553 Danish Children. *Pediatr Infect Dis J*. 2018;37(8):731-737. doi:10.1097/INF.0000000000001869
113. Brietzke E, Kauer Sant'anna M, Jackowski A, et al. Impact of childhood stress on psychopathology. *Braz J Psychiatry*. 2012;34(4):480-488. doi:10.1016/j.rbp.2012.04.009
114. Humphreys KL, Watts EL, Dennis EL, King LS, Thompson PM, Gotlib IH. Stressful Life Events, ADHD Symptoms, and Brain Structure in Early Adolescence. *J Abnorm Child Psychol*. 2019;47(3):421-432. doi:10.1007/s10802-018-0443-5
115. Donnelly JE, Hillman CH, Castelli D, et al. Physical Activity, Fitness, Cognitive Function, and Academic Achievement in Children: A Systematic Review. *Med Sci Sports Exerc*. 2016;48(6):1197-1222. doi:10.1249/MSS.0000000000000901
116. : Pem D. Factors Affecting Early Childhood Growth and Development: Golden 1000 Days. *Adv Practice Nurs* 2012;1:101. doi: 10.4172/2573- 0347.1000101
117. Waldfogel, J. , & Washbrook, E. V. (2010). Low income and early cognitive development in the UK: A report for the Sutton Trust. Sutton Trust. <http://www.suttontrust.com/public/documents/1Sutton_Trust_Cognitive _Report.pdf>
118. Olsson D, Mogren I, Forsberg B. Air pollution exposure in early pregnancy and adverse pregnancy outcomes: a register-based cohort study. *BMJ Open*. 2013;3(2):e001955. Published 2013 Feb 5. doi:10.1136/bmjopen-2012-001955
119. Rosen EM, Muñoz MI, McElrath T, Cantonwine DE, Ferguson KK. Environmental contaminants and preeclampsia: a systematic literature review. *J Toxicol Environ Health B Crit Rev*. 2018;21(5):291-319. doi:10.1080/10937404.2018.1554515
120. DIABIMMUNE Study Grp. No evidence of the role of early chemical exposure in the development of -cell autoimmunity. Environmental Science and Pollution Research, 2019;26(2), 1370-1378. <https://doi.org/10.1007/s11356-018-3659-6>
121. Perera FP, Rauh V, Whyatt RM, et al. Effect of prenatal exposure to airborne polycyclic aromatic hydrocarbons on neurodevelopment in the first 3 years of life among inner-city children. *Environ Health Perspect*. 2006;114(8):1287-1292. doi:10.1289/ehp.9084
122. HM Government. *An Evidence Review Of The Drivers Of Child Poverty For Families In Poverty Now And For Poor Children Growing Up To Be Poor Adults*. London: HM gov; 2014. https://assets.publishing.service.gov.uk/government/uploads/system/uploads/attachment_data/file/285389/Cm_8781_Child_Poverty_Evidence_Review_Print.pdf. Accessed December 3, 2020.
123. Grey, Tracy, Hári Sewell, Gillian Shapiro, and Fahmida Ashraf. Mental Health Inequalities Facing UK Minority Ethnic Populations: Causal Factors and Solutions. *Journal of Psychological Issues in Organizational Culture* 2013:3: 146–57
124. Campbell A. Getting it right for looked after children and young people. Gov.scot. https://www.gov.scot/binaries/content/documents/govscot/publications/strategy-plan/2015/11/getting-right-looked-children-young-people-strategy/documents/00489805-pdf/00489805-pdf/govscot%3Adocument/00489805.pdf. Published 2015. Accessed December 10, 2020.
125. [Fleming, M.](http://eprints.gla.ac.uk/view/author/46258.html) , Fitton, C. A., Steiner, M. F.C., McLay, J. S., Clark, D., King, A., [Mackay, D. F.](http://eprints.gla.ac.uk/view/author/3947.html)  and [Pell, J. P.](http://eprints.gla.ac.uk/view/author/7115.html)   Educational and health outcomes of children and adolescents receiving antiepileptic medication: Scotland-wide record linkage study of 766 244 schoolchildren. [*BMC Public Health*](http://eprints.gla.ac.uk/view/journal_volume/BMC_Public_Health.html), 2019:19, 595. (doi: [10.1186/s12889-019-6888-9](http://dx.doi.org/10.1186/s12889-019-6888-9)) (PMID:[31101093](http://europepmc.org/abstract/MED/31101093)) (PMCID:[PMC6525436](http://europepmc.org/articles/PMC6525436))
126. [Fleming, M.](http://eprints.gla.ac.uk/view/author/46258.html) , Fitton, C. A. , Steiner, M. F.C. , McLay, J. S. , Clark, D. , King, A. , [Lindsay, R.](http://eprints.gla.ac.uk/view/author/11507.html) , [Mackay, D. F.](http://eprints.gla.ac.uk/view/author/3947.html)  and [Pell, J. P.](http://eprints.gla.ac.uk/view/author/7115.html)  Educational and health outcomes of children treated for type 1 diabetes: Scotland-wide record linkage study of 766,047 children. [*Diabetes Care*](http://eprints.gla.ac.uk/view/journal_volume/Diabetes_Care.html), 2019:42(9), pp. 1700-1707. (doi: [10.2337/dc18-2423](http://dx.doi.org/10.2337/dc18-2423)) (PMID:[31308017](http://europepmc.org/abstract/MED/31308017)) (PMCID:[PMC6706279](http://europepmc.org/articles/PMC6706279))
127. [Fleming, M.](http://eprints.gla.ac.uk/view/author/46258.html) , Fitton, C. A., Steiner, M. F.C., McLay, J. S., Clark, D., King, A., [Mackay, D. F.](http://eprints.gla.ac.uk/view/author/3947.html)  and [Pell, J. P.](http://eprints.gla.ac.uk/view/author/7115.html)  Educational and health outcomes of children treated for asthma: Scotland-wide record linkage study of 683,716 children. [*European Respiratory Journal*](http://eprints.gla.ac.uk/view/journal_volume/European_Respiratory_Journal.html), 2019:54(3), 1802309. (doi: [10.1183/13993003.02309-2018](http://dx.doi.org/10.1183/13993003.02309-2018)) (PMID:[31196949](http://europepmc.org/abstract/MED/31196949)) (PMCID:[PMC6727030](http://europepmc.org/articles/PMC6727030))
128. [Fleming, M.](http://eprints.gla.ac.uk/view/author/46258.html) , Fitton, C. A., Steiner, M. F.C., McLay, J. S., Clark, D., King, A., [Mackay, D. F.](http://eprints.gla.ac.uk/view/author/3947.html)  and [Pell, J. P.](http://eprints.gla.ac.uk/view/author/7115.html)   Educational and health outcomes of children treated for attention deficit hyperactivity disorder: Scotland-wide record linkage study of 766,244 children. [*JAMA Pediatrics*](http://eprints.gla.ac.uk/view/journal_volume/JAMA_Pediatrics.html), 2017:171(7), e170691. (doi:[10.1001/jamapediatrics.2017.0691](http://dx.doi.org/10.1001/jamapediatrics.2017.0691)) (PMID:[28459927](http://europepmc.org/abstract/MED/28459927))
129. Chapple, S. (2009). Child well‐being and sole‐parent family structure in the OECD. OECD. <https://doi.org/10.1787/225407362040>
