# Supplementary figures and images for: Using graphic modelling to identify modifiable mediators of the association between area-based deprivation at birth and offspring unemployment
Source: PLoS One. 2021 Mar 31;16(3):e0249258. doi: 10.1371/journal.pone.0249258 (PMC8011734; doi:10.1371/journal.pone.0249258)

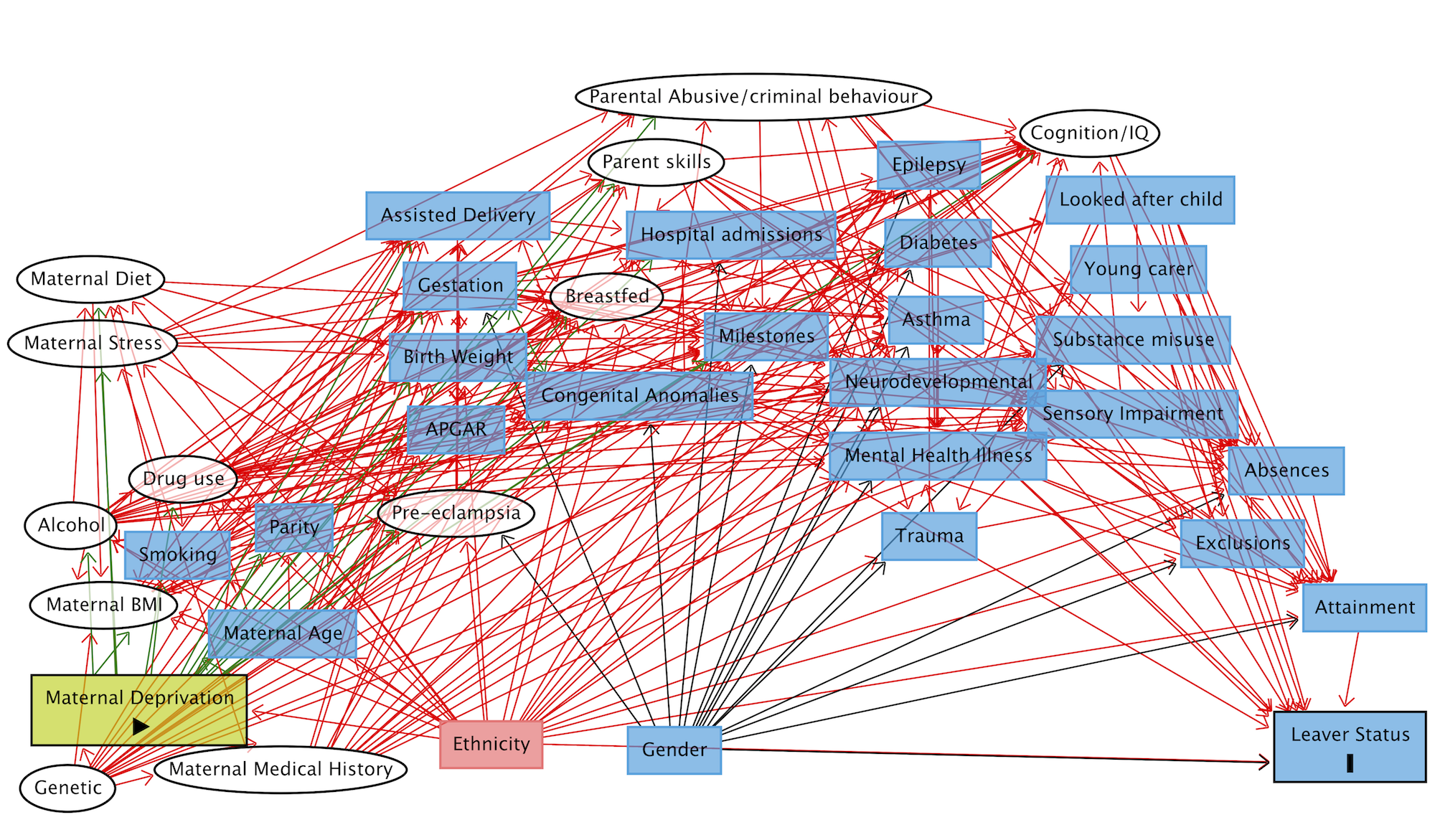

Supplement: S1 Fig — (TIF) [file pone.0249258.s003.tif]
